# Structural and diffusion weighted MRI demonstrates responses to ibrutinib in a mouse model of follicular helper (Tfh) T-cell lymphoma

**DOI:** 10.1371/journal.pone.0215765

**Published:** 2019-04-23

**Authors:** Rebecca L. Allchin, Michael E. Kelly, Sami Mamand, Anthony G. Doran, Thomas Keane, Matthew J. Ahearne, Simon D. Wagner

**Affiliations:** 1 Leicester Cancer Research Centre and Ernest and Helen Scott Haematology Research Institute, University of Leicester, Leicester, United Kingdom; 2 Core Biotechnology Services, University of Leicester, Leicester, United Kingdom; 3 European Bioinformatics Institute, Hinxton, Cambridge, United Kingdom; University of Toledo, UNITED STATES

## Abstract

Recent analyses of the genetics of peripheral T-cell lymphoma (PTCL) have shown that a large proportion of cases are derived from normal follicular helper (Tfh) T-cells. The sanroque mouse strain bears a mutation that increases Tfh cell number and heterozygous animals (Roquin^san/+^) develop lymphomas similar to human Tfh lymphoma. Here we demonstrate the usefulness of Roquin^san/+^ animals as a pre-clinical model of Tfh lymphoma. Long latency of development and incomplete penetrance in this strain suggests the lymphomas are genetically diverse. We carried out preliminary genetic characterisation by whole exome sequencing and detected tumor specific mutations in Hsp90ab1, Ccnb3 and RhoA. Interleukin-2-inducible kinase (ITK) is expressed in Tfh lymphoma and is a potential therapeutic agent. A preclinical study of ibrutinib, a small molecule inhibitor of mouse and human ITK, in established lymphoma was carried out and showed lymphoma regression in 8/12 (67%) mice. Using T2-weighted MRI to assess lymph node volume and diffusion weighted MRI scanning as a measure of function, we showed that treatment increased mean apparent diffusion coefficient (ADC) suggesting cell death, and that change in ADC following treatment correlated with change in lymphoma volume. We suggest that heterozygous sanroque mice are a useful model of Tfh cell derived lymphomas in an immunocompetent animal.

## Introduction

Peripheral T-cell lymphomas (PTCL) are a histologically, genetically and clinically heterogeneous group of diseases. ALK^+^ anaplastic large cell lymphoma (ALCL) has a 5-year overall survival of ~70% but for the other common PTCL subtypes (ALK^-^ ALCL, angioimmunoblastic T-cell lymphoma (AITL) and peripheral T-cell lymphoma not otherwise specified (PTCL-NOS)) the clinical outlook is poor (5-year overall survival ~35%) [1].

AITL is one of the two most common PTCL subtypes (~36%) [2], the other being PTCL-NOS, and has been a focus of interest because the malignant cells express CD10 [3], PD-1, CXCR5 and BCL6 [4], all markers characteristic of the normal CD4^+^ T-cell subset, follicular helper (Tfh) T-cells, which are found in germinal centers. Subsequent gene expression profiling studies confirmed that AITL and a subset of PTCL-NOS are likely to be derived from Tfh cells [5,6]. Tfh lymphoma has a characteristic genetic landscape with defects in epigenetic modifiers [7–10], RHOA [11,12] and some T-cell receptor proximal signalling molecules [13]. Recently the category nodal peripheral T-cell lymphoma with Tfh phenotype was introduced to include AITL, follicular T-cell lymphoma and some PTCL-NOS [14].

Normal Tfh cells are required for high affinity antibody production and are also essential for the development of autoimmunity in mouse models [15,16]. The sanroque strain was produced by systematic screening of mice for the production of anti-double-stranded DNA autoantibodies following chemical mutagenesis [17]. Analysis of this strain showed specific defects in Tfh cell regulation with increased size of germinal centres and Tfh cell numbers due to a point mutation in a ubiquitin ligase, Roquin, that regulates inducible co-stimulator (ICOS), a surface molecule with essential roles in T-cell activation. While animals homozygous (Roquin^san/san^) for the mutation develop enlarged spleen and lymph nodes and autoimmunity by 7-weeks [17], about 50% of heterozygous mice (Roquin^san/+^) develop a disease like AITL by 6-months [18]. Crossing heterozygous mice with animals deficient in CD28 or ICOS or SLAM associated protein (SAP) markedly reduced lymphoma incidence supporting the notion that Tfh cells are responsible for the development of disease.

Interleukin-2-inducible kinase (ITK) is expressed in human Tfh lymphoma [19]. ITK is specific for T-cells [20] and is essential for signalling from the T-cell receptor (TCR) [21,22] and chemokine induced migration [23,24]. Mice bearing homozygous disruptions of ITK show defects in CD4^+^ T-cell differentiation [25–29]. ITK is, therefore, an attractive target for treatment of Tfh lymphoma. ITK is a homolog of Bruton's tyrosine kinase, which is expressed in B-cells. Ibrutinib is a BTK/ITK inhibitor, which is in clinical use for the treatment of chronic lymphocytic leukaemia [30] and mantle cell lymphoma and is active against both human and mouse enzymes [31].

In this work we have carried out a preliminary genetic analysis of tumors developing in Roquin^san/+^ mice and, in order to provide evidence for introducing ibrutinib into clinical trials a pre-clinical study of this agent in Roquin^san/+^ mice, which had developed tumors, was carried out and responses were quantitated by structural (T2-weighted MRI) and functional imaging (diffusion weighted MRI).

## Methods

### Whole exome sequencing

DNA from a tumor and liver of 2 heterozygous mice, the liver from a heterozygous mouse with no tumor development at 8 months and the liver of a WT mouse was used. DNA was extracted from whole tumor containing both B- and T-cells. As described in detail previously [32] libraries were made using SureSelectXT Mouse All Exon Kit (Agilent Genomics) and sequenced on an Illumina HiSeq producing 75bp paired-end reads. Bioinformatic analysis was carried out using a previously described pipeline [32]. After removal of Illumina tag sequences and poor quality bases the sequencing reads from each sample were aligned to the C57BL/6 J GRCm38 (mm10) mouse reference genome using BWA-MEM (v0.7.5) with default parameters. Each BAM file (one per sample) was then sorted and filtered for possible PCR and optical duplicates using Picard Tools (v1.64). To improve SNP and indel calling, the GATK v3.0 ‘IndelRealigner’ tool, was used to realign reads around indels using default options. SAMtools (v 1.3.1) mpileup was used to generate VCF files and BCFtools (v 1.3.1) was used to filter the variants. Ensembl VEP tool version 88 was used to predict the effect of the filtered SNVs and Indels on gene transcripts. The anticipated mutation in the Roquin gene was detected in the tumor and constitutional DNA of mice genotyped as Roquin^San/+^ (S1 Fig) but not from the wild-type mouse sample. Mean coverage was 32x (range 18x to 46x) (S1 Table). Tumor and constitutional DNA from two Roquin^San/+^ mice (SRQ5293 and SRQ5301) were compared with a reference mouse sequence (C57BL/6J GRCm38 (mm10) mouse reference genome). After filtering there were variants in 13 genes in the SRQ5293 tumor sample, which were not found in germline DNA from the livers of a wild-type Roquin^+/+^ control sample or a Roquin^San/+^ mouse without tumor development and which passed the quality filters and were judged to be deleterious by SIFT (S2 Table) [33]. For tumor SRQ5301 variants were detected in 9 genes.

### Cell culture

CD4^+^ T-cells (1 x 10^5^ cells/well) were isolated using a CD4^+^ T-cell Isolation kit (130-104-454) (Miltenyi, Bergisch Gladbach, Germany) and cultured in a round-bottomed 96-well plate pre-coated with purified hamster anti-mouse CD3e (BD Biosciences, 553057) and hamster anti-mouse CD28 (BD Biosciences, 553294) for 72 hours. As described in detail previously [34] ATP luminescence was employed as a measure of cell viability/proliferation (CellTiter-Glo (CTG) Luminescent Cell Viability Assay (Promega, Madison, WI, USA). Luminescence was read using the Infinite 200 PRO (Tecan, Männedorf, Switzerland) with i-control software (Tecan).

### Western blot

Isolated primary mouse CD4^+^ T-cells, or single cell suspensions from whole tumor were lysed with radioimmunoprecipitation (RIPA) buffer (Tris-HCl pH 8.0 (50 mM), NaCl (150 mM) 1% sodium deoxycholate, and 0.1% SDS) supplemented with protease and phosphatase inhibitors (Sigma). Similarly the mouse B-cell lymphoma cell lines Bal-17, WEHI231 and A20 were also lysed in RIPA. Total cell lysates were incubated on ice for 10 to 15 minutes and centrifuged at 15000 x g for 10 minutes at 4°C to remove debris.

As described in detail previously [34] lysates were separated by SDS-PAGE on 7.5% Mini-protean TGX Precast gels (BioRad, Hercules, CA, USA), and transferred to a polyvinyldine difluoride membrane (Mini Format, 0.2 μM PVDF Single Application, BioRad). The blot was incubated in TBST (Tris-HCl pH 7.6 (20 mM), NaCl (136 mM), and 0.1% Tween-20) supplemented with 5% skimmed milk for 1 hour at room temperature. The membrane was incubated with primary antibody in blocking solution at 4°C overnight. Anti-ITK (ab32113, Abcam, Cambridge, UK) was used at 1:1000. Loading control was anti-GAPDH (1:10000) (#2118, Cell Signaling Technology, Danvers, MA, USA). After 3 to 5 washes with TBST, the blot was incubated with secondary antibody with shaking for 1 hour at room temperature. Secondary antibody used was anti-rabbit-IgG-HRP conjugated (1:2000) (#7074, Cell Signaling Technology).

Blots were washed five times for 10 minutes, and signal was detected with chemiluminescent HRP substrate (BioRad, Hercules, CA, USA) and imaged (SRX-101A X-Ray Film Processor, Konica Minolta, Bloxham Mill, UK) using medical X-ray film (Fujifilm, Tokyo, Japan).

### *In vivo* experiments

Mouse work was carried out at the Department of Biomedical Science (DBS) Preclinical Research Facility (PRF). The work was carried out under project licence 60/4371 and, subsequently project licence, P8E5F4055, which were granted following Home Office Review. The work was carried out in accordance with Home Office regulations and in line with the ARRIVE guidelines [35]. The work was ethically approved by the University of Leicester Animal Welfare Ethical Review Body (AWERB). Mice were housed in a clean environment and supplied with sterile food and water *ad libitum*. Sequencing demonstrated the previously reported mutation [18] (S1 Fig). During drug treatments mice were weighed 3 times a week, or more frequently if there were concerns about their condition, and soggy diet provided in cases of weight loss. Lymph nodes became palpable from about 4 months old. Palpable lymph nodes were measured in 2 dimensions using calipers three times a week. Following others [18] cervical, axillary, brachial, and inguinal lymph nodes were measured. Mice were determined to be tumor bearing if a lymph node from any area was palpable. No wildtype (Roquin^+/+^) animals developed tumors.

Ibrutinib was made up in a 10% 2-Hydroxypropyl-β-cyclodextrin (2-HBD) solution at a concentration of 25mg/ml and stored at -20°C. This was diluted to 2.5mg/ml in 1% 2-HBD for delivery by oral gavage at a dose of 25mg/kg each day. Vehicle treated mice received an equivalent volume of 1% 2-HBD.

### MRI scanning

As described in detail previously [36] MRI scanning was performed on a 9.4T Agilent scanner (Agilent Technologies, Santa Clara, CA, USA) with a 310mm bore diameter and 6cm inner-diameter gradient coil (1000mT/m maximum gradient strength). A 4cm millipede RF coil was used for RF transmission and reception. Physiological monitoring was achieved using a custom monitoring and gating system (SA Instruments, Stony Brook, NY, USA). Mouse body temperature was maintained at 37°C using a warm air fan and rectal temperature probe. Respiration was measured using a pneumatic pillow. All scans were performed during the light cycle under anaesthesia with 1–2% isoflurane in oxygen.

T2-weighted images were acquired using a respiratory-gated fast spin echo (FSE) sequence with TR/TE = 3000/40ms, 40 x 40mm field of view (256 x 256 matric), 32 x 0.8mm slices and two signal averages (scan duration = 6min 30secs). Axial slices of both the axillary and inguinal regions were acquired. In a subset of mice (n = 4 ibrutinib-treated mice), diffusion tensor imaging (DTI) of the inguinal region was performed. The DTI sequence used the same respiration-gated FSE sequence as the T2-weighted scan with TR/TE = 2000/26MS, 40 x 40mm field of view (128 x 128 matrix), 32 x 0.8mm slices. 14 diffusion encoding directions were used with a target b-value of 1000 s/mm^2^ (scan duration = 8min 30sec).

For volumetric analysis of lymph nodes, T2-weighted images were converted to DICOM format and analysed using region-of-interest tools in ImageJ Fiji (www.imagej.net)^10^. Apparent diffusion coefficient (ADC) maps were generated from DTI data using the diffusion analysis method in VnmrJ v.4.2 (Agilent Technologies, Santa Clara, CA, USA). Lymph node regions from the T2-weighted volume analysis were applied to ADC maps to calculate node-specific ADC values. Region of interest and ADC analysis was carried out by an experienced operator (MEK) who was blinded to the treatments the mice had received.

### Gene expression microarray

RNA was extracted from cell suspensions produced from the enlarged lymph nodes of treated and untreated mice. (RNeasy Mini Kit, Qiagen, Hilden, Germany). A total of 5 samples (3 ibrutinib treated and 2 untreated) were analysed using SurePrint G3 Mouse GeneExpression v2 Microarray Kit from Agilent. Samples selected were those with sufficient good quality RNA. The treated mice showed stable i.e. non-responsive, disease. One of the untreated mice showed progressive disease over the course of the treatment period (4 weeks) and the other showed stable disease. The microarray data are accessible through the Gene Expression Omnibus (GEO; GSE120402). Rank product analysis [37] was carried out using Microarray Experiment Viewer (MeV_4_8 version 10.2).

## Results

### Preliminary genetic characterisation of lymphomas in Roquin^San/+^ mice

In order to investigate the genetic abnormalities contributing to established lymphomas whole exome sequencing (WES) was carried out. Four genes (Hsp90ab1, Ccnb3, Rhoa and Pdzrn4) (Table 1) showed variants in one or both tumors as compared to Roquin^San/+^ without enlarged lymph nodes or Roquin^+/+^ animals (S2 Fig and S2 Table). Rhoa has an established role in human PTCL [11,12]. Hsp90ab1 is a member of the heat shock protein (HSP) family (19). HSPs have been found to be up-regulated in several cancer types although only a small proportion of haematological malignancy cases have been found to have a mutation in HSP90AB1 (20). Ccnb3 is a gene that has been associated with a subtype of bone sarcoma in which tumors containing a fusion between the BCL6 co-repressor (Bcor) gene and Ccnb3 gene have been shown to be distinct from Ewing sarcoma (21). Pdzrn4 has limited association with cancer although according to the COSMIC database mutations have been found in 0.4% of hematological malignancies (20).

**Table 1:** Tumor specific mutations. Mutations in four genes (Hsp90ab1, Rhoa, Ccnb3, Pdzrn4) showing changes from the reference sequence and not detected in constitutional DNA or in heterozygous (Roquin^san/+^) mice or wild-type littermates. Lymphomas from two mice (5293 and 5301) were sequenced. Both tumors showed mutations in Hsp90ab1, Ccnb3 and Pdzrn4 with identical mutations being found in Ccnb3 and Pdzrn4 in the two tumors. 5293 showed a mutation in Rhoa. The nucleotide and amino acid changes are indicated together with the respective variant allele frequency (VAF).

Tumor	Gene	cDNA	Amino acid	VAF (%)
5301	Hsp90ab1	1885A>T	Asn629Tyr	14
		1889C>T	Pro630Leu	15
		2015A>T	Asp672Val	21
		2035C>A	Arg679Ser	23
		2044C>T	Arg682Cys	29
	Ccnb3	354A>C	Asn75Glu	43
5293	Hsp90ab1	1810C>T	Arg604Trp	11
		1820A>C	Lys607Thr	16
	Ccnb3	354A>C	Asn75Glu	46
	Rhoa	575T>C	Phe25Leu	41
	Pdzrnf4	2376G>A	Arg705Gln	54

### Roquin^San/+^ mouse lymphomas express ITK

**Fig 1:**
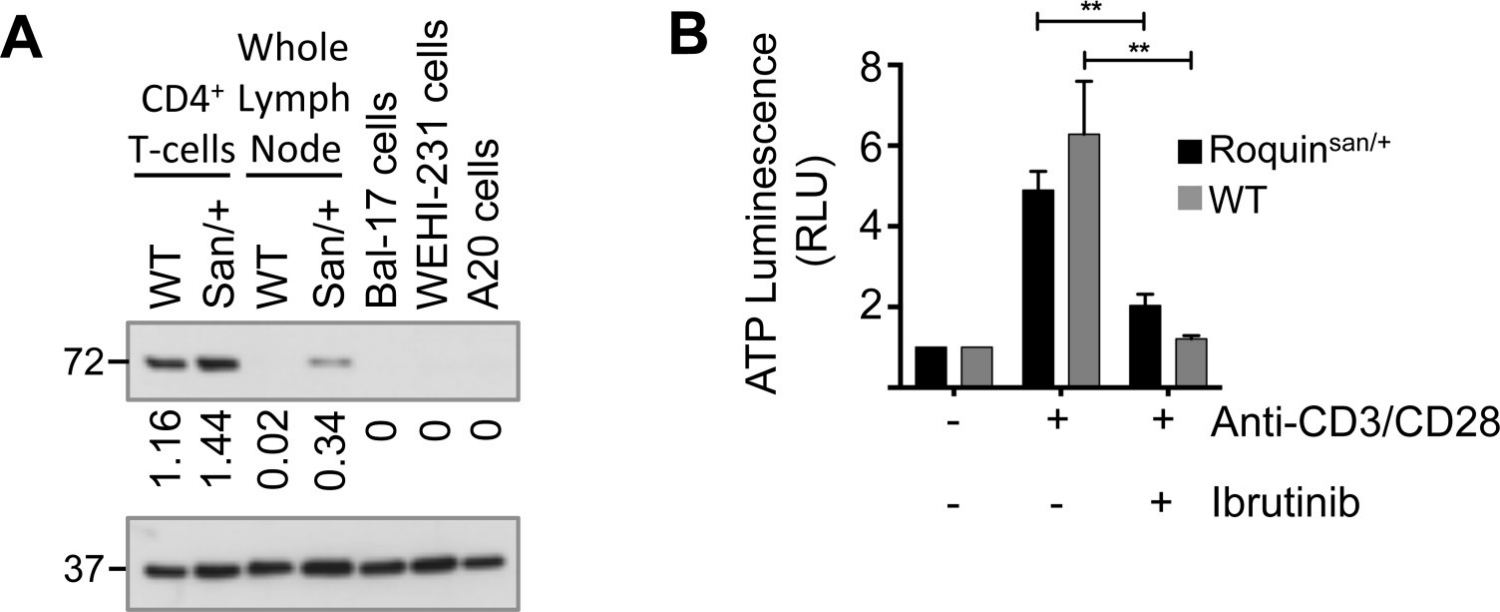
ITK expression in Roquin^san/+^ mice. **(A)** Western blot showing ITK expression in sorted CD4^+^ T-cells from wildtype (WT) or Roquin^san/+^ (San/+) and from whole lymph node. In order to demonstrate specificity of the antibody for mouse ITK and not BTK three mouse B-cell lines are included (Bal-17, WEHI231 and A20). GAPDH is loading control. **(B)** Purified splenic CD4^+^ T-cells from Roquin^san/+^ (black bars) and wild-type (grey bars) animals were stimulated with anti-CD3 and anti-CD28 antibodies in the presence or absence of ibrutinib. ATP luminescence is shown relative to that of unstimulated cells. Bars show mean±SEM. n = 7 Roquin^san/+^ and n = 10 wildtype. Ibrutinib caused significant (paired t-test) reduction in ATP luminescence (Roquin^san/+^ **P = 0.0016 and wildtype **P = 0.003).

The small molecule inhibitor, ibrutinib, is active against mouse ITK [31]. Western blots demonstrated that ITK was detectable and slightly more highly expressed in isolated CD4^+^ T-cells from Roquin^San/+^ mice than wild-type animals (normalised densitometry values 1.44 to 1.16) and there was also a marked difference in ITK expression in whole lymph node lysates (normalised densitometry values 0.34 to 0.02) (Fig 1A).

In order to establish responsiveness to ibrutinib *in vitro* isolated CD4^+^ T-cells were stimulated with anti-CD3 and anti-CD28 antibodies in the presence and absence of the drug (Fig 1B). ATP luminescence was not significantly different between stimulated Roquin^San/+^ and wild-type cells without ibrutinib but ibrutinib significantly (paired t-test) reduced levels for both Roquin^San/+^ (*P* = 0.0016) and wild-type T-cells (*P* = 0.003). This suggests that independent of genotype mouse CD4^+^ T-cells respond similarly to anti-CD3/CD28 and are similarly sensitive to ibrutinib.

### Some Roquin^San/+^ mouse lymphomas regress spontaneously

**Fig 2:**
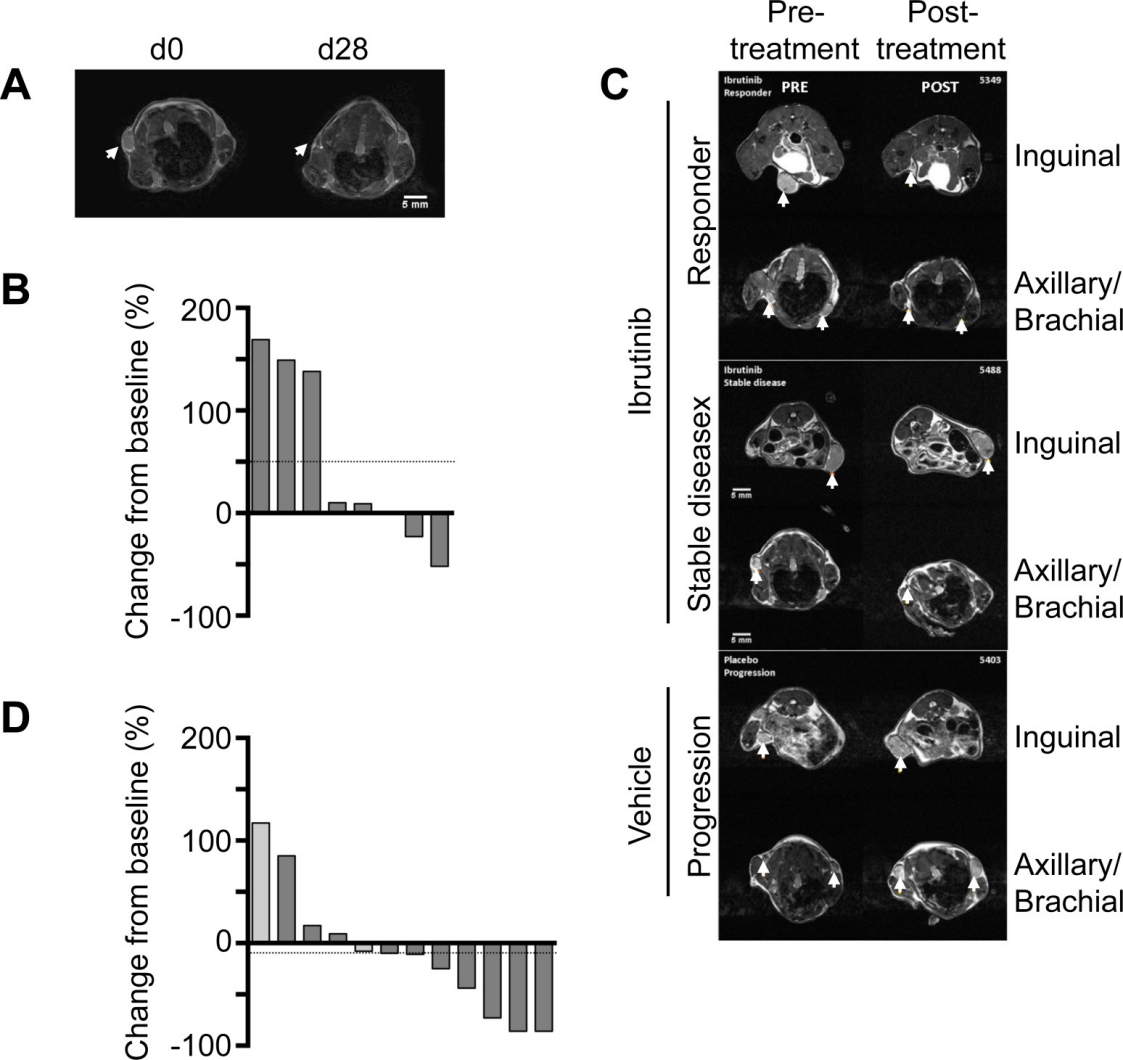
Ibrutinib causes repression of lymphoma growth. **(A)** T2 weighted MRI scans showing spontaneous regression and progression over the course of treatment with vehicle. **(B)** T2 weighted MRI scans showing exemplar slices from animals responding to ibrutinib or with stable disease. **(C)** Waterfall plot showing change in enlarged lymph node volume in mice treated with vehicle. Horizontal dotted line indicates mean change in lymph node size in the group of vehicle treated animals over the treatment period. n = 8. **(D)** Waterfall plot showing change in enlarged lymph node volume in mice treated with ibrutinib. Light grey columns indicate treatment with ibrutinib for 1 or 2 weeks and dark grey for 3, 4 or 7 weeks. Horizontal dotted line indicates mean change in lymph node size in the group of ibrutinib treated animals over the treatment period. n = 12.

We carried out a study in Roquin^San/+^ mice selected for palpable lymph nodes. Animals were either treated with vehicle (n = 8) or drug (n = 12). We noted spontaneous regression of enlarged lymph nodes in 2/8 (25%) of our group of animals treated with vehicle alone (Fig 2A and 2B and S3 Table). Tumor size was reduced from baseline volumes by 23% and 52% in these two animals. In three animals the enlarged lymph nodes were stable over the study period (change from baseline 10%, 9% and 1%) while in a further three mice lymph nodes increased in size (change from baseline 169%, 149% and 138%).

### Responses of Roquin^San/+^ mouse lymphomas to ibrutinib

Overall 8/12 (67%) of mice responded to ibrutinib with responses varying from 8% to 86% reductions in lymph node size (Fig 2C and 2D). In 4/12 (33%) of animals the enlarged lymph nodes failed to respond to ibrutinib, and showed an increase over the study period of 9% to 117%. The results are compatible with a modest effect of ibrutinib in limiting lymphoma development. In the group of mice treated with ibrutinib mean overall reduction in tumor size was 10%, but in the group of animals receiving vehicle alone mean increase in tumor size was 50%.

There was no significant (Mann-Whitney U-test) difference in lymph node size between vehicle 126.5 (64.5–209.8) mm^3^ (median and interquartile range) and treated groups 237.5 (95.8–316) mm^3^ before starting the experiment. However, at the end of the experiment although the median lymph node size of the treated group declined (134 (67–224) mm^3^) while that of the vehicle group rose (205 (56.25–229.5) mm^3^) there was no significant difference between these groups.

### Ibrutinib produces a gene signature with similarities to that observed in T-cells from Itk^-/-^ mice

Therefore, ibrutinib appeared to produce modest overall effects on tumor growth confounded by spontaneous regression. We considered the possibility that ibrutinib failed to achieve effects on gene expression in tumours that did not respond to the drug. Gene expression microarray was carried out and rank product analysis showed differences between ibrutinib and vehicle treated lymphomas (S4 Table). Analysis of the KEGG pathway T-cell receptor (TCR) genes showed up-regulation of many transcripts such as membrane Cd3 or signalling components Pik3 and Nfkb while a smaller number of transcripts were down-regulated by ibrutinib e.g. Il4 (S3 Fig). Analysis of transcript levels in Itk deficient mice have previously shown both up and down-regulated genes [38]. On the assumption that a small molecule ITK inhibitor will cause some of the gene expression changes observed in Itk deficient mice we compared fold changes in gene expression between ibrutinib and vehicle treated animals with the results of Blomberg et al. (2009). 23 upregulated transcripts were present on the microarray and of these 16 (70%) were also upregulated (i.e. ≥ 1.5 fold) in ibrutinib treated animals. Similarly 20 transcripts that were down-regulated in Itk deficient mice were present on the microarray and 10 (50%) were down-regulated (i.e. ≤ 0.7 fold) (Table 2).

**Table 2:** Comparison of fold change in transcript level between ibrutinib and vehicle treated animals with previously established transcriptional signatures from Itk deficient mice (Blomberg et al. 2009). 23 genes upregulated and 20 genes downregulated in CD3^+^ T-cells from Itk deficient mice are listed. Mean transcript levels of each genes in ibrutinib or vehicle treated animals are shown together with the fold change in level. The fold increases (≥ 1.5 fold) are colored red and the fold reductions (≤ 0.7 fold) blue.

	Gene	Mean Expression		Fold Change
		Ibrutinib	Vehicle	
**Sorted CD3**^**+**^ **T-cells—Upregulated**	CYBB	3.5	12.3	0.3
	ZEB2	6.2	13.5	0.5
	TGFBI	5.2	10	0.5
	PLEK	8.9	11.1	0.8
	ARHGAP24	3.1	3.6	0.9
	SOSTDC1	4.1	3.3	1.2
	GZMM	3.2	2.5	1.3
	CLEC1B	4.4	2.7	1.6
	KLRA3	3.3	2.0	1.7
	FABP4	3.7	2.0	1.8
	AMY1	4.5	2.0	2.3
	PSP	5.6	2.5	2.3
	CENPK	7.4	3.1	2.4
	KLRA7	5.2	2.2	2.4
	FCER1G	7.1	2.7	2.6
	TUBB1	6.2	2.2	2.8
	CDH13	10.7	3.7	2.9
	SIRPB1	6.9	2.0	3.5
	ZBTB16	9.8	2.8	3.6
	KLRA8	11.6	2.6	4.5
	TMCC2	12.9	2.4	5.4
**Sorted CD3**^**+**^ **T-cells—Downregulated**	CNKSR2	2.8	8.7	0.3
	MS4A4D	2.7	8.1	0.3
	ARMC9	4.9	11.2	0.4
	CALM3	3.1	6.7	0.5
	COPS3	8.0	12.1	0.7
	RAB3IP	5.5	7.9	0.7
	LRRC19	6.4	9.0	0.7
	ACTN2	8.1	11.8	0.7
	OASL2	7.4	10.2	0.7
	EID3	9.4	13.1	0.7
	CNN3	7.2	8.7	0.8
	SLC6A19	11.8	14	0.8
	EIF2S3X	3.8	4.3	0.9
	AMPD1	3.7	3.8	1.0
	UPP2	4.1	4.1	1.0
	TRPM1	5.7	4.3	1.3
	GGT	5.7	4.3	1.3
	NEUROD4	6.4	4.0	1.6
	PDLIM4	6.5	4.0	1.6
	WASF1	7.8	4.1	1.9

### Assessment of response by diffusion weighted MRI

**Fig 3:**
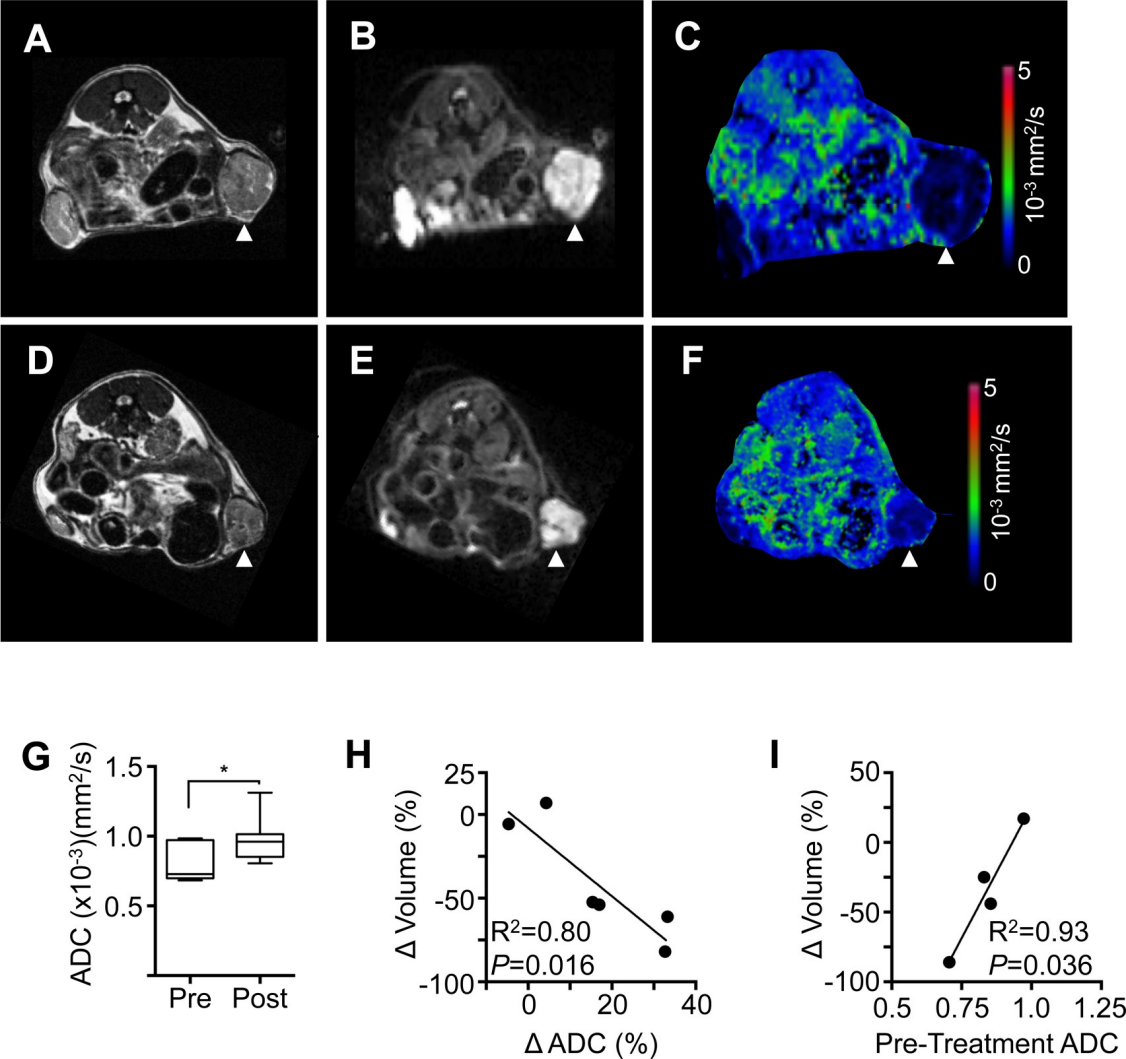
Functional imaging: Change in ADC correlates with change in lymphoma volume. Pre-treatment MRI scans from a representative mouse showing **(A)** T2-weighted, **(B)** diffusion-weighted and **(C)** ADC map. Corresponding post-treatment MRI showing **(D)** T2-weighted, **(E)** diffusion-weighted and **(F)** ADC map. **(G)** Treatment with ibtrutinib resulted in a significant increase in ADC (paired t-test, *P* < 0.05). **(H)** Change in lymph node volume after treatment (Δ Volume) measured by T2-weighted MRI and change in ADC after treatment (Δ ADC) measured by diffusion-weighted MRI were inversely correlated (R^2^ = 0.80, *P* = 0.016). n = 6 lymph nodes in 4 mice. **(I)** Change in lymph node volume after treatment (Δ Volume) correlates with pre-treatment ADC (R^2^ = 0.93, *P* = 0.036). n = 4 lymph nodes in 4 mice.

In order to provide functional information on the effects of ibrutinib on the state of tissues before and after treatment diffusion weighted MRI scanning was carried out (Fig 3A to 3F). Apparent diffusion coefficient (ADC) a relative measure of the diffusion of water within tissues, altered significantly with treatment (Fig 3G, paired t-test, *P* = 0.023). Furthermore, the change in ADC following treatment was found to correlate linearly with the change in lymph node volume (Fig 3H) (R^2^ = 0.8, *P* = 0.0161) indicating that decreasing node volume correlates with increasing ADC. Pre-treatment ADC was also found to correlate with the change in lymph node volume (Fig 3I) (R^2^ = 0.93, P = 0.036) indicating that elevated ADC prior to treatment may result in a reduced volumetric response to treatment.

## Discussion

There is interest in using patient derived xenograft (PDX) mouse models in order to promote the rational introduction of new therapies for PTCL [39]. At present there are relatively small numbers of PTCL PDXs but this approach will be important for future studies. Other potential mouse models of PTCL for pre-clinical testing are deficient in Tet2, either with the Rhoa^G17V^ [40] or without the mutation [41], or bear a translocation between ITK and spleen tyrosine kinase (SYK), a chromosomal abnormality, which is found in a small minority of AITL [42]. There is, therefore, no standard model system and yet recent advances have suggested new agents such as ITK or PI3Kδ inhibitors or anti-ICOS antibodies might be therapeutically useful [43]. We chose to assess the usefulness of heterozygous sanroque mice as a model system for preclinical testing. The advantage of this model is that it is a source of genetically diverse primary mouse lymphomas, as suggested by the long latency of development, incomplete penetrance and confirmed by the preliminary sequencing studies presented in this report, on a background that is immunocompetent. Intriguingly we noted spontaneous regression of some enlarged lymph nodes and we wonder whether this reflects a feature of Tfh lymphomas that has been described in human disease [44].

In order to begin to understand the genetic diversity of the Roquin^san/+^ mouse model we carried out a limited WES study comparing lymphoma DNA to constitutional DNA of the same mouse and the DNA of wild-type animals. We found a novel Rhoa mutation at residue 25 in one tumor (p.Phe25Leu) suggesting that this may co-operate with the Roquin-1 mutation to drive malignant Tfh proliferation. There is clear evidence for genetic intra-tumor heterogeneity in human PTCL: TET2 is mutated in a greater proportion of cases than RHOA and inspection of the published VAF results suggests sub-clonal variation in some cases [11,12]. In addition non-malignant B-cells have been found to bear TET2 mutations [45]. The VAFs we found are too high to be consistent with mutation in the Tfh population alone (~1% in Roquin^san/+^ mice [18]) but would be compatible with the total CD4^+^ T-cell population being involved (20 to 30% in Roquin^san/+^ mice [18]). Although unlikely in this mouse model mutations might also be present in both B- and T-cell populations. Other causes for the relatively high VAFs are also possible e.g. homozygous mutations or copy number variation, might occur in some cases. Further studies will be required to understand the genetics of mouse Tfh lymphoma.

We carried out gene expression profiling to attempt to demonstrate that a signature similar to that found in sorted CD3^+^ T-cells from Itk^-/-^ mice [38] was observed in ibrutinib treated whole lymphomas. We were not able to obtain sufficient numbers of sorted cells from this material for microarray analysis. This approach is potentially able to investigate drug effects in responding and non-responding lymphomas, which may produce candidate biomarkers, but sufficient material of high enough quality from tumors responding to ibrutinib could not be obtained. Therefore, while gene expression studies are capable of determining genetic correlates of response to ibrutinib they are limited by the technical difficulties of biopsy and the very small amount of viable tissue that may be remaining at the end of the experiment in responding tumors. In this paper we investigated *in vivo* transcriptional changes due to ibrutinib by comparing our experimentally derived data with published gene expression changes due to disruption of the *Itk* locus [38]. It is anticipated that, although there will be overlap between these two gene sets, they will not overlap due to off-target effects of the small molecule. This represents a limitation to our approach but the data we obtained supports ibrutinib as showing some effects on transcript levels *in vivo* similar to those seen in Itk deficient mice.

A recent phase 1 clinical study has explored ibrutinib in relapsed or refractory T-cell lymphoma [46]. Cases were not selected for ITK expression and, while the feasibility of using ibrutinib in this clinical setting was demonstrated there was only limited clinical efficacy. In order to obtain pre-clinical evidence for the use of ibrutinib in the setting of T-cell lymphoma that expresses ITK we carried out this study. Ibrutinib is also an attractive agent because it may have effects both on the malignant T-cell population and the non-malignant B-cells, which make up the majority of the tumor bulk.

We employed MRI scanning to assess lymph node size before and after treatment. Tumor size is often assessed using calipers to measure dimensions and then estimating volume. Although these methods are fast, inexpensive and do not require anaesthesia, they are highly operator-dependent, ineffective at measuring small, early stage tumors and unsuited to orthotopic and metastatic tumor models [47]. Imaging methods such as ultrasound, micro-CT, bioluminescence imaging, PET and MRI have all been shown to provide tumor volumes with increased accuracy, precision and reproducibility compared to caliper measurements [47–50].

In addition to tumor volume, MRI has the advantage that it can provide a multi-parametric assessment of tumor tissue characteristics by using methods such as diffusion- and perfusion-weighted imaging to provide parameters reflecting cell proliferation, apoptosis and vascularity, without requiring injection of contrast agent or substrates [51–53]. Diffusion-weighted MRI is a particularly promising method for imaging cell death after therapy. An increase in the ADC of water following treatment has been correlated with necrosis and also morphological changes associated with apoptosis such as cell shrinkage [54,55]

A significant increase in ADC measured by diffusion-weighted MRI was detected in a subset of ibrutinib-treated mice (Fig 3). Diffusion-weighted MRI has been suggested as a highly useful technique for assessment of lymphoma in patients [56,57]. An increase in tumor ADC following intervention or treatment of cancer is generally attributed to cell necrosis or apoptosis within the tumor, leading to reduced cell density, increased intracellular space and a concomitant reduction in the restriction of water diffusion provided by cell membranes and organelles [58,59]. Correlation of diffusion-weighted MRI results with alternative methods such as positron emission tomography further suggests that ADC values can act as functional biomarkers of treatment response [49]. We made the intriguing observation that pre-treatment ADC measurement appeared to predict response to ibrutinib (Fig 3I). This needs confirmation in a larger study but suggests that ADC might be a biomarker in this mouse model of T-cell lymphoma.

Overall, the study demonstrates that a subgroup of genetically diverse T-cell lymphomas in the Roquin^San/+^ immunocompetent mouse model respond to ibrutinib and preliminary data suggests that diffusion-weighted MRI may be predictive of response to this agent. The imaging approach we describe could be incorporated into clinical trials of novel compounds to treat T-cell lymphoma, while the mouse model and combined diffusion and T2-weighted MRI shows a methodology for pre-clinical testing of novel compounds.

## Supporting information

S1 Fig Detection of the Roquin-1 mutation. IGV screen view showing the nucleotide in Roquin-1 (Rc3h1) mutated in the sanroque strain (red arrowheads). While the mutated allele is not found in the wild-type mouse (WT control) it is present in a Roquin^san/+^ animal and in both lymphoma and constitutional DNA from two mice with tumors (5293 and 5301). For the Roquin^san/+^ control animal and the two mice with lymphomas the mutant allele is indicated by red shading and the wild-type allele by brown shading.
(TIF)

S2 Fig Whole exome sequencing. Analysis of WES data showed tumor specific mutations in four genes. The location of mutations in Hsp90ab1, Ccnb3, Rhoa and Pdzrn4 are shown. Green lollipops indicate that the mutation was detected in mouse 5301 and blue lollipops mutations found in mouse 5293. For Ccnb3 an identical mutation was found in lymphomas from mice 5293 and 5301 (grey lollipop). Numbering beneath each gene refers to amino acid position.
(TIF)

S3 Fig Hierarchical clustering of transcript levels of KEGG pathway T-cell receptor (TCR) signalling genes. Whole lymphoma from ibrutinib (n = 3) and vehicle (n = 2) treated mice were subjected to microarray analysis. Transcript levels of TCR signalling genes are shown.
(TIF)

S1 Table Whole exome sequence coverage. Whole exome sequence coverage at Chr1: 160,940,825 (exon 5 of the Roquin gene (Rc3h1) is shown in the five Roquin^san/+^ samples and the wildtype (WT control) mouse. Mean coverage is 32x (range 18x to 46x). The coverage at the wildtype allele and mutant allele are presented.
(XLSX)

S2 Table Genes showing mutations in lymphoma and constitutional DNA from two animals SRQ5293 and SRQ5301. Whole exome sequence results. Genes presented were mutated in lymphoma (Tumour) DNA or constitutional DNA from two animals 5293 and 5301. However these mutations were not present in DNA from wild-type mice or a Roquin^san/+^ animal without lymphoma development.
(XLSX)

S3 Table Lymph node volumes. Lymph node volumes (mm^3^) in mice treated with ibrutinib or control and percentage change over the course of treatment.
(XLSX)

S4 Table The most up-regulated and down-regulated transcripts in a Rank Product analysis of ibrutinib and vehicle treated tumors. RefSeq identity and gene name are shown with the *P*-value (MeV_4_8 version 10.2).
(XLSX)
