# Supplementary figures and images for: Structural and diffusion weighted MRI demonstrates responses to ibrutinib in a mouse model of follicular helper (Tfh) T-cell lymphoma
Source: PLoS One. 2019 Apr 23;14(4):e0215765. doi: 10.1371/journal.pone.0215765 (PMC6478326; doi:10.1371/journal.pone.0215765)

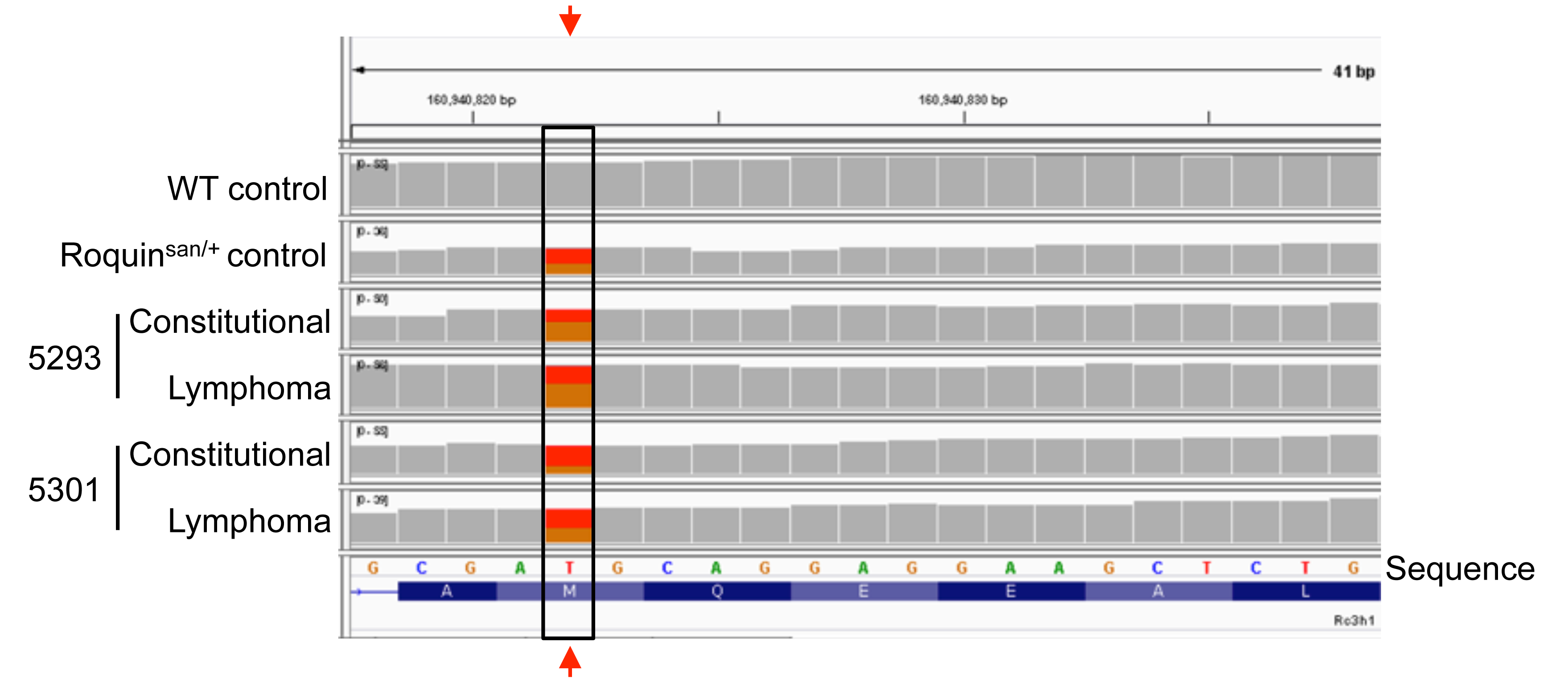

Supplement: S1 Fig — IGV screen view showing the nucleotide in Roquin-1 (Rc3h1) mutated in the sanroque strain (red arrowheads). While the mutated allele is not found in the wild-type mouse (WT control) it is present in a Roquinsan/+ animal and in both lymphoma and constitutional DNA from two mice with tumors (5293 and 5301). For the Roquinsan/+ control animal and the two mice with lymphomas the mutant allele is indicated by red shading and the wild-type allele by brown shading. (TIF) [file pone.0215765.s001.tif]

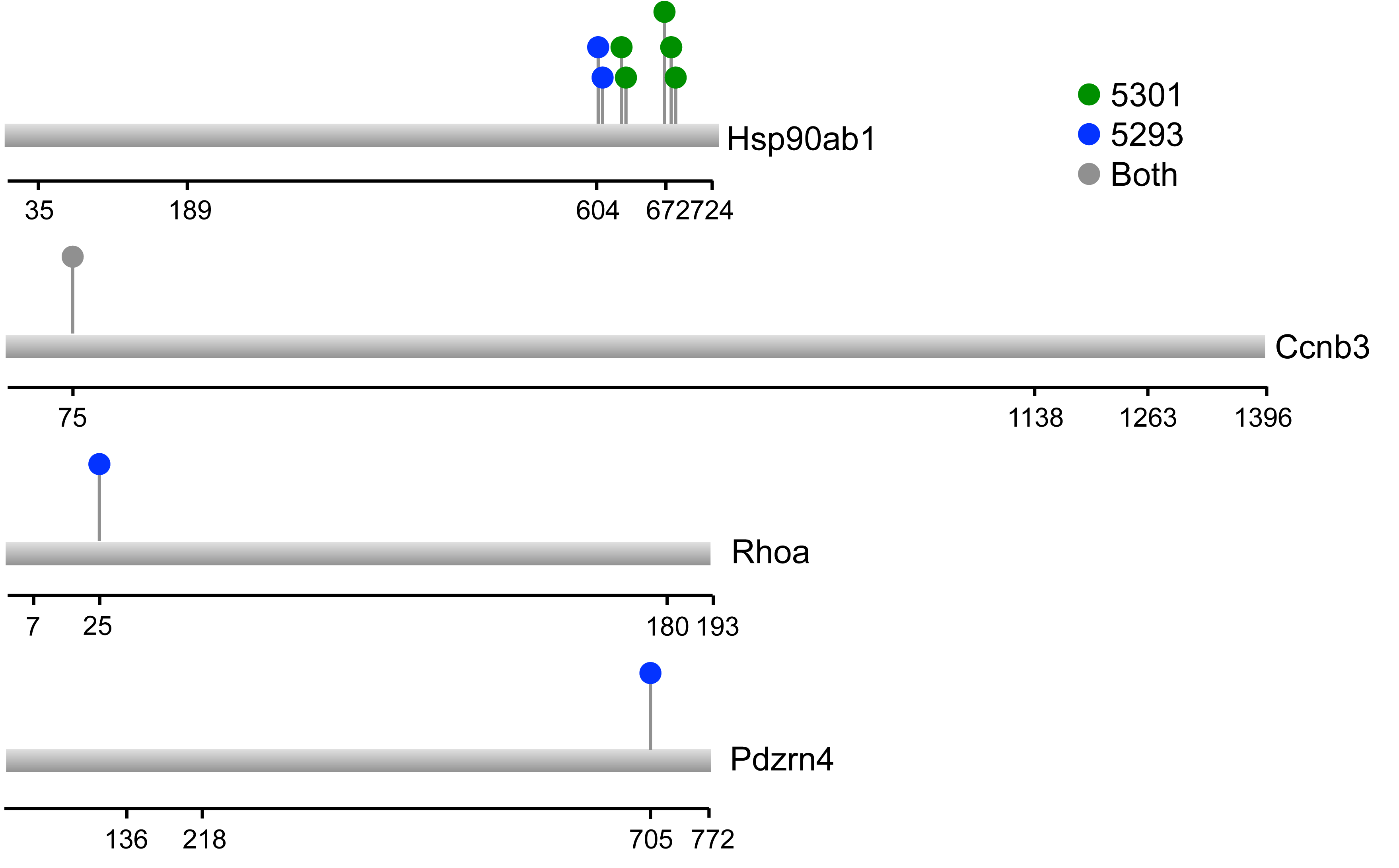

Supplement: S2 Fig — Analysis of WES data showed tumor specific mutations in four genes. The location of mutations in Hsp90ab1, Ccnb3, Rhoa and Pdzrn4 are shown. Green lollipops indicate that the mutation was detected in mouse 5301 and blue lollipops mutations found in mouse 5293. For Ccnb3 an identical mutation was found in lymphomas from mice 5293 and 5301 (grey lollipop). Numbering beneath each gene refers to amino acid position. (TIF) [file pone.0215765.s002.tif]

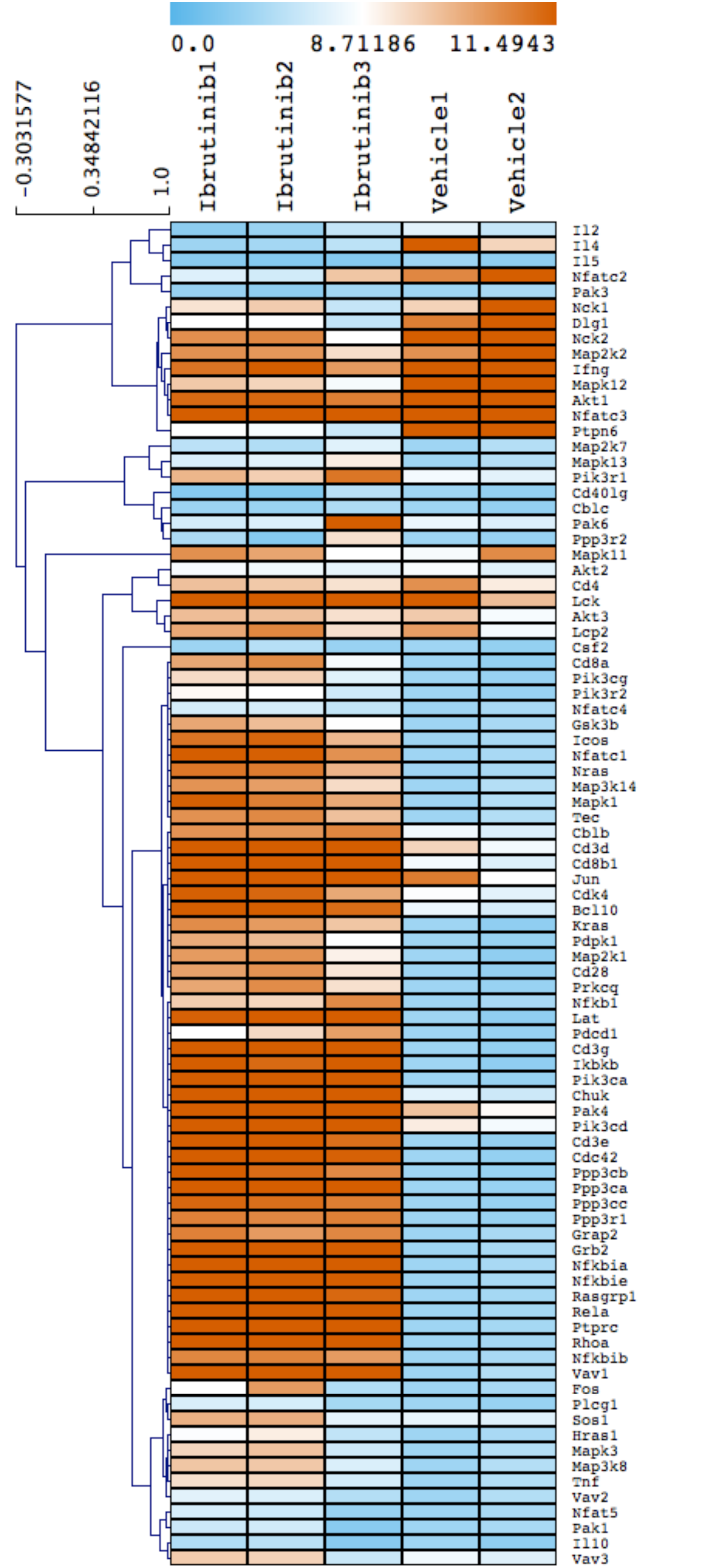

Supplement: S3 Fig — Whole lymphoma from ibrutinib (n = 3) and vehicle (n = 2) treated mice were subjected to microarray analysis. Transcript levels of TCR signalling genes are shown. (TIF) [file pone.0215765.s003.tif]
